# Recombination Events in Putative Tail Fibre Gene in *Litunavirus* Phages Infecting *Pseudomonas aeruginosa* and Their Phylogenetic Consequences

**DOI:** 10.3390/v14122669

**Published:** 2022-11-29

**Authors:** Marcin Górniak, Aleksandra Zalewska, Agata Jurczak-Kurek

**Affiliations:** Department of Evolutionary Genetics and Biosystematics, University of Gdańsk, Wita Stwosza 59, 80-308 Gdańsk, Poland

**Keywords:** recombination, phylogeny, *Litunavirus* phages, tail fibre gene, *Pseudomonas aeruginosa*

## Abstract

Recombination is the main driver of bacteriophage evolution. It may serve as a tool for extending the phage host spectrum, which is significant not only for phages’ ecology but also for their utilisation as therapeutic agents of bacterial infections. The aim of this study was to detect the recombination events in the genomes of *Litunavirus* phages infecting *Pseudomonas aeruginosa*, and present their impact on phylogenetic relations within this phage group. The phylogenetic analyses involved: the whole-genome, core-genome (*Schitoviridae* conserved genes), variable genome region, and the whole-genome minus variable region. Interestingly, the recombination events taking place in the putative host recognition region (tail fibre protein gene and the adjacent downstream gene) significantly influenced tree topology, suggesting a strong phylogenetic signal. Our results indicate the recombination between phages from two genera *Litunavirus* and *Luzeptimavirus* and demonstrate its influence on phage phylogeny.

## 1. Introduction

Viral genomics is nowadays a rapidly developing field. With the growing number of complete genomes in the database, the viral taxonomy is vastly dynamic and undergoing regular updates by the International Committee on Taxonomy of Viruses (ICTV). In 2020 a new family, *Schitoviridae*, was proposed within the order of tailed bacterial viruses (bacteriophages, phages) by Witmann et al. [1].

At present the family includes eight subfamilies, forty-eight genera and seventy-six species of bacteriophages infecting the classes of alpha–, beta– and gammaproteobacteria. A typical member of the family is *Escherichia* phage *N4*, the first isolate, currently assigned to the genus *Enquatrovirus* [1]. The genus of our interest in this study is *Litunavirus* within the *Mingulavirinae* subfamily of *Schitoviridae*, comprising 23 members, 10 of which are assigned to three species: *Litunavirus PA26*, *Litunavirus Ab09* and *Litunavirus LIT1*, while the rest remain unassigned (Table 1). According to the current rules introduced by ICTV, the criteria by which different phage genera and species are distinguished are established by an appropriate Study Group and may include natural and experimental host range and the degree of relatedness of their genomes or genes. Wittman and colleagues used 95% and 70% nucleotide sequence identity over the length of the genome as species and genus demarcation criteria, respectively [1]. Apart from that, all litunaviruses reveal podoviral morphology with icosahedral capsids of about 60–85 nm attached to 10–40 nm long tails at the portal vertex. Their genomes consist of linear double-stranded DNA of 72–73.3 kb, encoding three DNA-dependent RNA polymerases, including large (~1000 aa) virion-associated RNA polymerase (vRNAP), injected into the infected bacterium together with phage DNA, which is characteristic for N4-like phages.

*Litunavirus* phages infect *Pseudomonas aeruginosa*, a Gram-negative bacteria ubiquitous in soil, water, and on plants but also frequently found in clinical specimens as a human and animal opportunistic pathogen. In humans the bacterium is the main cause of community- or hospital-acquired infections of wounds, urinary tract, respiratory tract and bacteremia, among others. One of the factors that contributes to its pathogenicity is the ability to form antibiotic-resistant biofilms [2]. Due to the inapplicability of standard drugs for the destruction of *P. aeruginosa* biofilm, an alternative approach such as phage therapy utilising bacteriophages to eradicate pathogenic bacteria has been considered by researchers [3].

Some *Litunavirus* phages have been recently investigated as candidates in the phage therapy of animals. For instance, the therapeutic potential of phages YH30 and YH6 to treat hemorrhagic pneumonia in mink was evaluated both in mink and murine models [4,5]. The antibacterial efficacy of the phage PEV2 as a potential cure for canine otitis was assessed using a *Galleria mellonella* larvae model [6]. Other authors focused on eradicating the biofilm-forming strains of *Pseudomonas aeruginosa* by means of phage cocktails, especially in patients with cystic fibrosis [7]. Designing a broad-range phage cocktail, in general, is fundamental for its successful therapeutic use. One such approach is the ‘Appelmans protocol’ used by Eastern European researchers to generate therapeutic phages with novel lytic host ranges. Recently, Burrowes and colleagues developed a modification of this protocol in order to investigate the mechanisms of phage evolution in the cocktail. The authors selected a recombinant phage of significantly extended host spectrum in comparison with parent phages. Recombination traces in tail fibre protein genes were observed much more frequently than in the other regions of phage genomes, suggesting adaptive pressure on these structures [8].

Our results are in accordance with the observation that recombination in bacteriophages occurs frequently in genome regions putatively engaged in host recognition, and suggest the intergeneric recombination between two phage genera: *Litunavirus* and *Luzseptimavirus*. We identified the hypervariable genome region that involves the tail fibre protein gene and the adjacent downstream gene, and studied its impact on the phylogeny of *Litunavirus* phages. In the absence of the variable region in the analysis, the phylogenetic tree topology resembled that of the core-genome tree, which suggests a strong phylogenetic signal of the region. Several *Litunavirus* phages appeared to acquire the tail fibre gene from the other genus, which encourages us to question the species concept in phage classification.

**Table 1:** The summary information of *Litunavirus* and *Luzseptimavirus* phages analysed in this study.

Phage Name	Phage Origin	Phage Species	GenBank Number	Genome Size (bp)	BLASTn Results (KX171209.1 as Query)		Capsid Size (nm)	Tail Length (nm)	Tail Structures	Reference
					Query Coverage (%)	Sequence Identity (%)				
vB_Pae575P-3	Gdańsk (Poland)	UC	KX171209.1	72,728	100	100.00	60	30	nd *	[9]
vB_Pae1396P-5	Gdańsk (Poland)	UC	KX171210.1	72,508	100	99.96	60	30	nd *	[9]
PA26	Naju City (South Korea)	*Litunavirus PA26*	NC_041907.1	72,321	98	95.86	-	-	- **	[10]
PAP02	Seoul (South Korea)	UC	MT080102.1	73,345	99	95.63	-	-	-	[11]
YH30	Beijing (China)	*Litunavirus PA26*	KP994390.1	72,192	99	96.06	65	40	tail fibre-like structures ***	[4]
vB_PaeP_PYO2	Milan (Italy)	UC	MF490236.1	72,697	97	96.17	72	18	nd	[7]
vB_PaeP_DEV	Milan (Italy)	*Litunavirus Ab09*	MF490238.1	72,697	97	96.10	72	18	nd	[7]
RWG	Lubbock, Texas (U.S.)	*Litunavirus Ab09*	KM411958.1	72,646	94	97.36	-	-	-	[8]
PEV2	Olympia, Washington (U.S.)	*Litunavirus Ab09*	KU948710.1	72,697	94	97.34	70	30	nd	[12]
vB_PaeP_C2-10_Ab09	Indénié-Djuablin (Ivory Coast)	*Litunavirus Ab09*	HG962375.1	72,028	94	97.05	70	-	nd	[13]
ph_P3P1	Paris (France)	UC	LT594787.1	72,778	95	96.92	74	-	nd	[14]
vB_PaeP_TUMS_P121	Tehran (Iran)	UC	MZ955867.1	73,001	97	96.93	-	-	-	[15]
vB_PaeS_TUMS_P81	Tehran (Iran)	UC	OL519844.1	73,167	97	96.94	-	-	-	[16]
DL64	Bath (UK)	*Litunavirus LIT1*	KR054032.1	72,378	94	96.69	65	12–13	fibres protruding from capsid	[17]
YH6	Changchun (China)	UC	KM974184.1	73,050	95	96.55	65	25	nd	[5]
LY218	Alexandria Creek, Alabama (U.S.)	UC	MN906996.1	73,083	95	96.18	84–85	10–12	neck with collar	[18]
phi176	Lubbock, Texas (U.S.)	*Litunavirus Ab09*	KM411960.1	73,048	95	96.03	-	-	-	[8]
Pa2	Lubbock, Texas (U.S.)	*Litunavirus Ab09*	NC_027345.1	73,008	96	96.00	-	-	-	[8]
VB_PaeS_VL1	Nakhon Pathom (Thailand)	UC	OK665488.1	73,308	97	95.74	-	-	-	[19]
LP14	Qingdao (China)	UC	MH356729.1	73,080	97	95.63	60	30	tail fibre-like structures ***	[20]
vB_PaeP_MAG4	Puławy (Poland)	UC	KR052142.1	72,979	94	97.59	63	36	collar and ring-like structure with appendages	[21]
LIT1	Leuven (Belgium)	*Litunavirus LIT1*	FN422399.1	72,544	95	96.82	70	30	nd	[12]
CMS1	Stanford, California (U.S.)	UC	OM937766.1	72,673	97	96.11	-	-	-	[22]
vB_PaeS_TUMS_P6	Tehran (Iran)	*Luzseptimavirus KPP21*	OL519842.1	73,885	17	85.47	-	-	-	[23]
vB_PaeS_TUMS_P10	Tehran (Iran)	*Luzseptimavirus KPP21*	OM782452.1	74,200	17	85.60	-	-	-	[24]
vB_Pae_AM.P2	Kollam, Kerala (India)	*Luzseptimavirus KPP21*	MT416090.1	73,308	17	85.37	-	-	tail fibre-like structures ***	[25]
KPP21	Kochi City (Japan)	*Luzseptimavirus KPP21*	LC064302.1	73,420	18	84.91	-	-	tail fibre-like structures ***	[26]
LUZ7	Leuven (Belgium)	*Luzseptimavirus LUZ7*	FN422398.1	74,901	12	78.82	70	30	nd	[12]

UC—unclassified *Litunavirus*; “-” no data available; “nd”—not detected; *—data unpublished; **—authors described phage as *Myoviridae*; ***—our interpretation of other authors’ electron microphotograph.

## 2. Materials and Methods

Whole-genome sequences of 23 members of the *Litunavirus* genus and 5 members of the *Luzseptimavirus* genus were downloaded from National Center for Biotechnology Information (NCBI) (Bethesda, MD, USA). All bacteriophage strains used are listed in Table 1. Sequences were selected based on the highest similarity with the two *Pseudomonas aeruginosa* phages isolated and described earlier in our laboratory [9]: vB_Pae575P-3 (GenBank: KX171209.1) and vB_Pae1396P-5 (GenBank: KX171210.1), determined by the BLASTn algorithm, using default options. 94% to 100% of query coverage with >95 percent of sequence identity hits were taken for *Litunavirus* genus determination, and 12% to 18% of query coverage with sequence identity of 78% to 86% for *Luzseptimavirus*.

The Recombination Detection Program version 5.3 (RDP5.3) (http://web.cbio.uct.ac.za/~darren/rdp.html, accessed on 1 June 2022) was used to detect possible recombination regions in phage genomes [27]. A putative recombination event was flagged when detected by all seven methods: RDP [28], GENECONV [29], MaxChi [30], BootScan [31], SiScan [32], Chimaera [33], and 3Seq [34].

Based on the received results, four strategies were carried out to investigate the phylogenetic position of studied phages within the *Litunavirus* genus: 1. whole-genome analysis; 2. core-genome analysis; 3. variable region analysis; 4. whole-genome minus variable region analysis. The core genome analysis involved gene coding for seventeen N4-like proteins conserved in the *Schitovridae* family (Table S1), described elsewhere [1]. Variable region analysis considered the putative tail fibre protein gene, particularly 4 out of 5 fragments corresponding to protein domains (GrpE, GDSL-like lipase/acylhydrolase, pyocin knob, C-terminal end) and the adjacent downstream region including ribosome binding sites, and gene coding for the hypothetical protein. In phages vB_Pae575P-3 and vB_Pae1396P-5 the variable regions involve ORFs 53, 52 and ORFs 52, 51, respectively. Detailed information about the variable region composition and positions in the studied matrix is presented in Table S2.

The progressive alignment algorithm implemented in Mauve version 20150226 (https://darlinglab.org/mauve/download.html, accessed on 1 June 2022) [35] was used to align all genomes. Unaligned, unique fragments (characteristic only for one record) were excluded from the analyses. The core protein coding genes and variable regions were downloaded from each accession separately, concatenated into core and variable matrices, respectively, and translated into amino acids. Seaview 4 was used to align these protein matrices. Additionally, each domain of the variable region, as well as each core gene, were analysed separately. Each core gene tree (17 bootstrap consensus trees > 95 BS with P-distance and corrected distance based on DNA model) was used to generate consensus network using SplitsTree6 version 0.03a (https://software-ab.cs.uni-tuebingen.de/download/splitstree6/welcome.html, accessed on 1 June 2022) [36]. A model of DNA evolution for each core gene matrix was estimated using JModelTest [37].

To show the direction of recombination within a variable region, the Autumn Algorithm method [38] with default options was used to construct a hybridization network in SplitsTree6 [36]. The hybridization network was based on two incongruent bootstrap consensus trees >95 BS. In each analysis, “whole-genome minus variable region tree” was analysed versus tail fibre protein gene fragments corresponding to the 4 protein domains described above.

All matrices were analysed using PAUP* (Phylogenetic Analysis Using Parsimony and Other Methods) version 4.0a (https://paup.phylosolutions.com/, accessed on 1 June 2022) [39]. The optimality criterion was set to p-distance using the Neighbour-Joining algorithm (NJ) with default options. The robustness of the tree topology was assessed by bootstrap analyses based on 1000 replicates.

## 3. Results

### 3.1. Recombination Detection

**Figure 1:**
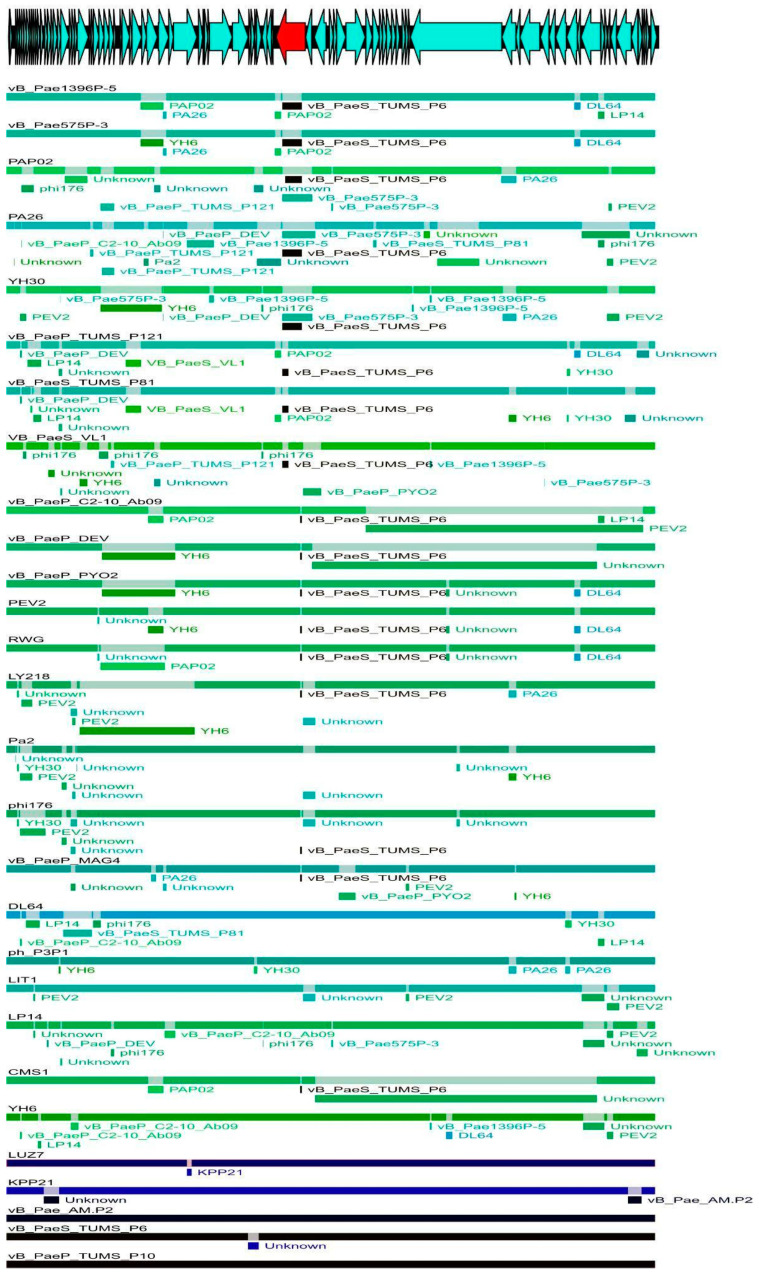
The map of recombination events within the genomes of *Litunavirus* and *Luzseptimavirus* bacteriophages. Phage genomes are depicted as coloured lines: light blue and green for *Litunavirus*; dark blue and black for *Luzseptimavirus*. The recombinant fragments of the genomes are marked by rectangles in the colours corresponding to the putative source phage genomes. The schematic genome map of the studied phages is present at the top of the figure, and the variable region is marked with a red arrow.

The analysis of the whole-genome matrix using The Recombination Detection Program (RDP) software indicated many recombination events within *Litunavirus* (Figure 1). One region in particular drew our attention. Recombination in the gene coding for tail fibre protein (3200 nt) and the adjacent downstream gene (195 nt) was present in almost all *Litunavirus* phages. Moreover, as depicted in Figure 1, for most litunaviral phages the variable region seems to have originated from *Luzseptimavirus* vB_PaeS_TUMS_P6, which suggests intergeneric recombination. The variability observed within the tail fibre protein gene considered each gene fragment corresponding to a particular protein domain separately, suggesting distinct recombinatorial paths. The analysis of the core-genome matrix also indicated many recombination events within *Litunavirus* (Figure 1 and Figure S1). The recombination was also confirmed by the incongruence of the core-genome tree topology shown as a consensus network (Figure S1).

### 3.2. Phylogenetic Analysis

**Figure 2:**
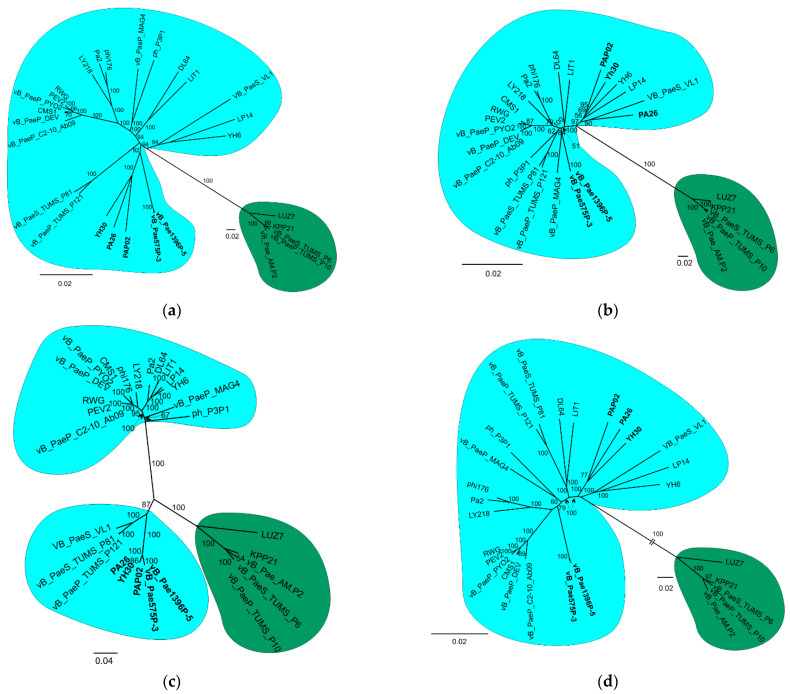
Four strategies of *Litunavirus* phylogenetic analysis: (**a**) whole-genome tree; (**b**) core-genome tree; (**c**) variable region tree; (**d**) whole-genome minus variable region tree. *Litunavirus* and *Luzseptimavirus* phages are circled in blue and green, respectively. Bootstrap support values are presented on branches, and phylogenetic distance scale is shown as a bar for the whole graph (**c**) or distinct clades in its proximity (**a**,**b**,**d**). The most crucial viral taxa are presented in bold.

The whole-genome (strategy 1) and the core-genome (strategy 2) analyses supported monophyly of the *Litunavirus* and *Luzseptimavirus* genera with high bootstrap support (BS) (Figure 2a,b). However, the trees’ topology was significantly different for three *Litunavirus* phages: YH30, PA26 and PAP02. In the whole-genome tree, these phages clustered with the phages vB_Pae575P-3 and vB_Pae1396P-5 with BS 92 (Figure 2a), while in the core-genome tree they clustered with phages YH6, LP14 and VB_PaeS_VL1 with BS 97 (Figure 2b). In the variable region tree (Figure 2c), the genus *Litunavirus* was not monophyletic. Two groups of *Litunavirus* phages were observed, one of which clustered with *Luzseptimavirus* with high bootstrap support (BS 100) (Figure 2c). In this group phages PA26, YH30, PAP02, vB_Pae575P-3 and vB_Pae1396P-5 formed one subgroup, and phages pVB_PaeS_VL1, vB_PaeS_TUMS_P81 and vB_PaeS_TUMS_P121 formed the other. Both subgroups together were supported with BS 87. Interestingly, the exclusion of the variable region from the whole-genome matrix resulted in a tree topology (Figure 2d) that was consistent with the core-genome tree (Figure 2c), suggesting a strong phylogenetic signal generated by the presence of the variable region in the phage genomes. Both the whole-genome minus variable region tree and the core-genome tree reflect ancestor–descendant phylogenetics.

**Figure 3:**
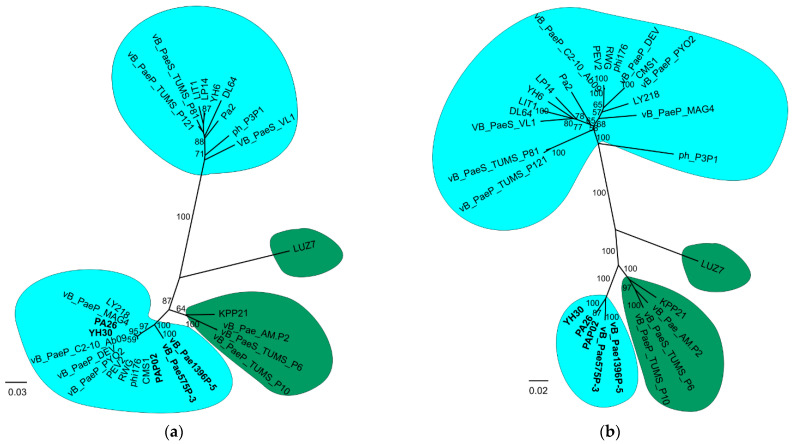

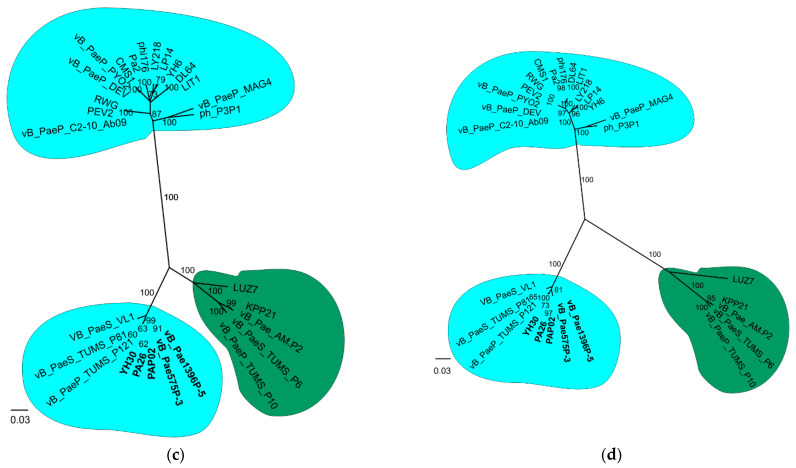
Phylogenetic analysis of particular protein domains within the variable region: (**a**) GrpE-like domain tree; (**b**) GDSL-like lipase/acylhydrolase domain tree; (**c**) pyocin knob domain tree; (**d**) C-terminal end tree. *Litunavirus* and *Luzseptimavirus* phages are circled in blue and green, respectively. Bootstrap support values are presented on branches and the phylogenetic distance scale is shown as a bar for particular graphs in its proximity. The most crucial viral taxa are presented in bold.

The separate analyses of particular protein domains in the variable region showed various tree topologies (Figure 3). Although in all domain trees, the *Litunavirus* genus was divided into two groups, one of which always clustered with the *Luzseptimavirus* genus, the phage composition of these groups differed between trees. Interestingly, several *Litunavirus* phages, YH30, PA26, PAP02, vB_Pae575P-3 and vB_Pae1396P-5, were present in the group clustering with *Luzseptimavirus* phages in each domain tree, suggesting that intergeneric recombination involved the whole variable region in these phages (Figure 3). In some phages, like pVB_PaeS_VL1, vB_PaeS_TUMS_P81 and vB_PaeS_TUMS_P121, the recombination occurred in two out of the four domains (Figure 3c,d), while in other phages it occurred only in one domain (Figure 3a). The composition of the *Luzseptimavirus* clade was also diverse. In the GrpE-like tree and GDSL-like tree, the LUZ7 phage was a sister to the rest of the *Luzseptimavirus* phages, constituting a separate species, *KPP21* (Figure 3a,b). The topologies of the pyocin knob domain tree and C-terminal end tree were almost identical. However, visual inspection of the alignments of these domains showed different patterns, so we decided not to combine them into one matrix. This is highlighted by the different distances between three major clades on the phylogenetic trees. Bacteriophage LUZ7 in these trees clustered together with other luzseptimaviruses and recombinant litunaviruses (Figure 3c,d).

### 3.3. Hybridization Network

**Figure 4:**
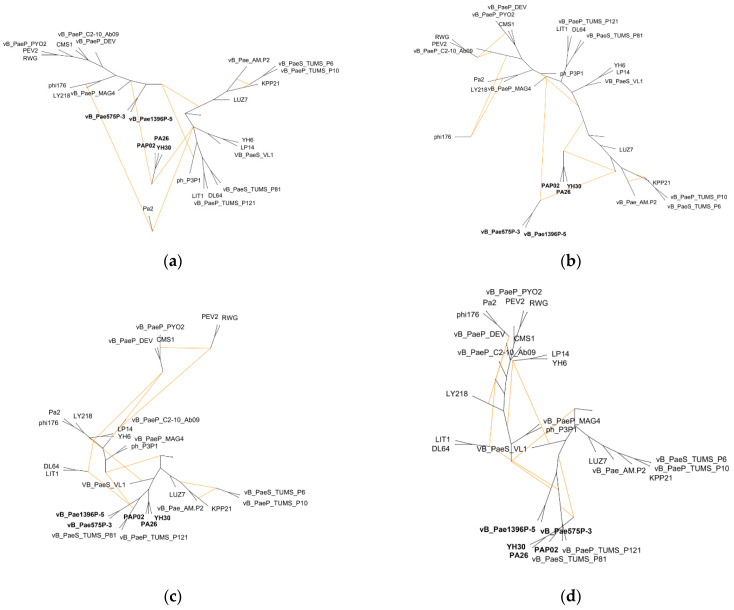
Hybridization network analysis. Each conflicted pair of trees involves the whole-genome minus variable region tree and one of the domain trees: (**a**) GrpE tree; (**b**) GDSL-like lipase/acylhydrolase tree; (**c**) pyocin knob domain tree; (**d**) C-terminal end tree. Reticulate nodes formed by orange lines indicate the recombinant phages. The most crucial viral taxa are presented in bold.

The application of the Autumn algorithm enabled the presentation of the conflict between the genome minus variable region tree (ancestor–descendant phylogenetics) and putative tail fibre gene domain trees as hybridization/recombination networks. The Autumn algorithm minimises the number of reticulations in the computed network and does not require the input trees to be bifurcating [38]. In Figure 4, we presented four pairs of conflicted trees (genome minus variable region tree vs. GrpE tree/GDSL-like lipase tree/pyocin knob domain tree/C-terminal end tree). The analyses gave rise to a representative set of three, six, twelve and nine hybridization networks, respectively. In general, all the networks from each analysis were similar, therefore only one network from each analysis was shown (Figure 4a–d). The networks contained four (Figure 4a), six (Figure 4b), six (Figure 4c), and five (Figure 4d) reticulate nodes, respectively. Each node indicated a recombination event. Intra– and intergeneric recombination can be observed in each network. Our results indicate that recombination of the putative tail fibre protein gene (variable region) first took place in the ancestor of *Litunavirus* phages of European and American origin and has intergeneric characteristics (Table 1 and Figure 4a). This recombination was also unidirectional from *Luzseptimavirus* to *Litunavirus* (Figure 4a). As a result, all descendants received the recombinant GrpE domain region. Exceptionally, two European phages vB_Pae1396P-5 and vB_Pae575P-3 also kept the remaining three domains of the tail fibre gene (Figure 4b–d). Similar observations were noted for three *Litunavirus* phages of Asian origin: YH30, PA26 and PAP02, which suggests that recombination in these viruses might have occurred independently (Figure 4a,b).

## 4. Discussion

### 4.1. Phylogenetic Position of Litunavirus Phages

Horizontal gene transfer (HGT) has proven to be the major driving force of bacteriophage evolution [8,40]. The molecular mechanisms leading to HGT consist of nonhomologous and homologous recombination, both found in bacteriophages. Nonhomologous recombination requires little or no sequence homology between recombining DNAs and occurs randomly across the genome, disrupting genes and gene blocks [40]. Temperate phages, such as λ, ∅80 or Mu, are capable of site-specific recombination, an example of nonhomologous recombination, to integrate their genomes into host chromosomes during lysogenic infection [41]. Homologous recombination occurs more frequently, requires high homology between sequences, and is promoted by the phage recombination machinery [40]. It can occur when two or multiple phages coinfect the same bacterial cell (superinfection), which is still a poorly understood phenomenon. Coinfection may consider obligately lytic phages that replicate simultaneously, and appears to be prevalent in the natural bacterial population [42]. The other scenario is when a prophage embedded in the host genome is engaged in gene shuffling with a coinfecting lytic virus. As a result, many phage genomes appear as genetic mosaics composed of exchangeable “modules” of single or multiple genes [40]. However, several viral species including temperate (λ) and exclusively lytic phages (T4) are found to have evolved mechanisms to prevent superinfection, such as superinfection exclusion that may disturb the adsorption or DNA injection of a subsequently infecting phage [43]. Recently, Hunter and Fusco have been investigating this phenomenon in a stochastic agent-based computational model [43]. They drew the conclusion that superinfection exclusion may exert negative effects on the long-term adaptation of a viral population, but in the short term it gives the phages the opportunity to overtake a superinfecting population even if the latter has a much higher growth rate [43].

In this study we demonstrated the impact of recombination events on the phylogeny of *Litunavirus* phages (Figure 1). A standard phylogenetic analysis involves the construction of a phylogenetic tree, a diagram that depicts the lineages of evolutionary descent of different species from a common ancestor. However, such an approach has a major drawback: it does not reflect the complex evolutionary relationship between bacteriophages driven by HGT, which was highlighted recently by Dion and colleagues [40]. We agree that, consequently, the whole-genome phylogenetic analysis does not show the entire evolutionary history of the phages studied. However, on one hand, if the analysis involves closely related phages derived from a common ancestor (for example, within a genus), the whole-genome phylogenetic tree can be a good indicator of the kinship of these biological entities. On the other hand, one must be aware that some internal nodes in such a phylogenetic tree are artifacts and do not indicate a common ancestor. This is the case when a recombinant fragment (from a different genus) is independently introduced into different closely related evolutionary lineages (these lineages are often called species in the case of phages). The same effect is also obtained when the recombinant fragment is transmitted by intrageneric recombination. Paradoxically, as a result of HGT, the most closely related phages (with the lowest genetic distance) are not necessarily derived from a common ancestor. If the recombinant fragment is a small fraction of the genome, its influence on whole-genome based classification may be negligible. However, if the recombination stretches over large fractions of the genome, it may be significant.

Such a case is presented in our analyses. Both the whole-genome minus variable region tree (Figure 2d) and the core-genome tree (Figure 2b) probably reflect ancestor–descendant phylogenetics. A recombinant fragment containing a significant part of the tail fibre protein gene and the adjacent downstream gene, representing less than 5 percent of the entire genome, apparently derived from the genus *Luzseptimavirus* and detected in five *Litunavirus* phages, namely vB_Pae1396P-5, vB_Pae575P-3, PAP02, PA26 and YH30, significantly influenced their phylogenetic position within the *Litunavirus* genus (Figure 2a,c,d). As a result, phages became more closely related to one another (Figure 2a) while possibly not sharing a common ancestor, and were more distant from other members of the genus (Figure 2b), which suggests that the phylogenetic signal strength of the recombinant fragment is strong (Figure 3). In the case of phages in which the recombination has occurred in one or two out of the four domains of the tail fibre gene, the phylogenetic position of these phages was not changed. The approach to classification may also differ significantly in the case of phages representing taxa of different degrees of variability. The impact of HGT events on phylogeny is inversely proportional to the variability of the recipient phage species.

Our results indicated that, if the construction of phylogenetic trees/classification of phages are based on the whole-genome analysis (genomic similarities), phage variability and the impact of HGT events on phylogeny should be assessed. Exclusion of HGT fragments that have strong phylogenetic impact should allow ancestor–descendant phylogeny to be estimated (Figure 2d). In addition, to determine the complexity of HGT, a hybridization/recombination network should be generated. As we show in Figure 4, in the case of multidomain proteins, each domain may have different phylogeny.

### 4.2. Bacteriophage Species Concept

Our research demonstrates that the classification of viruses to particular species is highly unstable and dependent on horizontal gene transfer (HGT) events. The probable acquisition of a new tail fibre protein gene region through homological recombination by some *Litunavirus* phages from *Luzseptimavirus* phages has drastically changed their position in the whole-genome phylogenetic tree, which is most evident for phages PAP02, PA26 and YH30 (Figure 2a,b).

According to the International Committee on Taxonomy of Viruses (ICTV) a viral species is the lowest taxonomic level in the hierarchy, defined as a monophyletic group of viruses whose properties can be distinguished from those of other species by multiple criteria such as host range and the degree of relatedness of viral genomes (95% of identity for *Litunaviruses*). At the moment, there are three species in the *Litunavirus* genus: *LIT1*, *Ab09* and *Pa26*. Following this key, based on our genomic analysis, about eight to twelve species should be distinguished. Because multiple monophyletic clades at different depths can be extracted from a single tree, this approach is very subjective and not informative. We therefore suggest basing the phage species concept on gene flow through homologous recombination. This approach for defining phage and virus species corresponds to the Biological Species Concept (BSC) that has been defining members of a biological species by their ability to exchange genetic material for over 75 years [44]. BSC originally excluded acellular organisms (viruses and bacteriophages), reproducing clonally, like other asexual organisms, from species-level classification because clonal individuals are reproductively isolated from one another. Nevertheless, as proposed by Bobay and Ochman, BSC definition can possibly be applicable to cellular and acellular lifeforms, including bacteriophages, due to sufficient levels of gene flow enabling biological species to be distinguished [44]. Based on the authors’ assumptions and our results received in this study, we suggest regarding the whole genus of *Litunavirus* as one biological species, and particular phylogenetic lineages which have obtained variable RBP domains through recombination may be differentiated analogically to bacterial strains, in case of the confirmed sharing of mutually adaptive features.

### 4.3. Speculations on RBS

In the course of our research we have made some speculations on the location of receptor binding sites (RBSs) in *Litunavirus* as the recombinant fragments detected in phage genomes considered the region coding for putative tail fibre protein. In general, RBSs of tailed phages are located in the tail structures. Many phages with long-tailed virions, e.g., coliphage T4, use their long tail fibres [45]. Some phages with short tails employ both tail tube and tail fibres (coliphage T7), or tail spikes (*Salmonella* phage P22) [46,47]. Other phages of podoviral morphology, like the N4 coliphage belonging to *Schitoviridae*, use a sheath surrounding the tail tube for adsorption to the host receptor. The mature virion of N4 lacks the tail fibres but possesses 12 appendages projecting from the neck connecting the head and tail [48].

Unlike their relative, the N4-like *Pseudomonas* phages belonging to *Litunavirus* analysed in this study lack the genes coding for the tail tube sheath. Moreover, the cluster of N4 tail genes in litunaviral genomes is apparently replaced by a cluster of genes embedded in the replication module, as described earlier [12]. Interestingly, five different tail proteins are encoded in the *Litunavirus* genomes: tail fibre protein, lytic tail fibre protein, tail fibre protein J, tail protein, and tail assembly protein. However, electron microscopy analyses confirmed the presence of tail fibre-like structures only in four *Litunavirus* phages: YH30, DL64, LP14, and vB_PaeP_MAG4; and in two *Luzseptimavirus* phages used as an outgroup in this study: vB_Pae_Am.P2 and KPP21 (Table 1). Some additional tail structures, like a collar, were also present (Table 1).

In this study, recombination events were observed within the gene region coding for the largest tail fibre protein gene (over 3000 nt) and the adjacent downstream gene coding for hypothetical protein (195 nt) (Table S2 and Figure 1). Tail fibre protein in the phages studied consists of five putative domains: N-terminal end, GrpE domain, GDSL-like lipase/acylhydrolase, pyocin knob domain and C-terminal end. The four latter domains revealed recombinatorial character (Figure 4) and seem to be attributed to host cell recognition and entry. Using each domain separately in phylogenetic analysis revealed various tree topologies (Figure 3a–d), suggesting multiple recombination events within the region and thus diverse evolutionary paths. GrpE proteins are the adenine nucleotide exchange factor of DnaK (Hsp70)-type ATPases. In *Escherichia coli* it is a part of the chaperone machine (the DnaK/DnaJ/GrpE complex) that performs key cellular functions, e.g., de novo protein folding and targeting to biological membranes, protein quality control, assembly or disassembly of oligomeric complexes, and cell signalling [49]. The GrpE domain present in phage tail fibres may be involved in protein folding. The requirement for chaperones for the proper trimeric assembly of tail fibres has previously been reported for T4 coliphage [45]. GDSL-like lipases are putatively involved in the degradation of bacterial surface components, enabling the entrance and/or exit of the host cell [50]. Finally, pyocin knob domains are found in saccharide-binding tail fibres and tail spikes and are likely to be involved in host cell recognition and binding. Interestingly, high-molecular-weight R-type pyocins are bacteriocins produced by some strains of *Pseudomonas aeruginosa* specifically to kill other strains of the same species [51]. Such a characteristic of tail fibres’ protein domains appears to indicate their role in host recognition, which is particularly important for developing successful phage therapy strategies to combat multidrug-resistant *Pseudomonas aeruginosa* infections with *Litunavirus* phages.

## 5. Conclusions

Our findings prove the occurrence of intergeneric recombination between phages from two genera within the *Schotiviridae* family: *Litunavirus* and *Luzseptimavirus*. The phenomenon significantly impacts litunaviral phylogenetic position, suggesting that phage classification at the level of species is controversial and perhaps should be neglected. The analysed recombination fragment involved the region coding for a putative tail fibre protein gene, most likely engaged in bacterial host recognition. The genetic variability of the region may lead to host spectrum extension or cause host specificity switching, which will be of interest in our further research. The identification of highly variable region domains of tail fibre protein may also guide future engineering of the studied phages.

## Data Availability

The data presented in this study are openly available in FigShare at https://doi.org/10.6084/m9.figshare.21196228 (accessed on 23 September 2022).

## Supplementary Materials

The following supporting information can be downloaded at: https://www.mdpi.com/article/10.3390/v14122669/s1, Table S1: The list of genes coding for seventeen N4-like proteins conserved in *Schitovridae* family with their position in the genome of selected *Litunavirus*, vB_Pae1396 P-5, used in phylogenetic core-genome analysis in this study; Table S2: The composition of the variable region used in the phylogenetic analyses in this study and its position in the alignment and in the genome of the selected *Litunavirus*, vB_Pae1396P-5; Figure S1: (a) The map of recombination events within core genomes of *Litunavirus* and *Luzseptimavirus* bacteriophages. Phage core genomes are depicted as coloured lines. The recombinant fragments of the genomes are marked by rectangles in the colours corresponding to the putative source phage genome. (b) The 95% Neighbour Joining bootstrap consensus network based on the P-distance of the core-genome tree of *Litunavirus* phages. (c) The 95% Neighbour Joining bootstrap consensus network based on the Model-distance of the core-genome tree of *Litunavirus* phages.
