# Supplementary material for: Recombination Events in Putative Tail Fibre Gene in Litunavirus Phages Infecting Pseudomonas aeruginosa and Their Phylogenetic Consequences
Source: Viruses. 2022 Nov 29;14(12):2669. doi: 10.3390/v14122669 (PMC9786124; doi:10.3390/v14122669)
Supplement: Supplementary file 1 [file viruses-14-02669-s001.zip › Table S1.pdf]

Table S1. The list of genes coding for seventeen N4-like proteins conserved in *Schitovridae* family with their position in the genome of selected *Litunavirus*, vB_Pae1369P-5, used in phylogenetic core-genome analysis in this study.

	**Phage N4 Product**	**vB_Pae1369P-5 Protein Accession Number**	**Gene coordinates**
1	RNA polymerase small subunit	ANT44388.1	KX171210.1: 5843-6775
2	RNA polymerase large subunit	ANT44392.1	KX171210.1: 7678-8919
3	ATPase	ANT44403.1	KX171210.1: 13787-14857
4	gp25	ANT44405.1	KX171210.1: 15387-16616
5	DNA polymerase	ANT44408.1	KX171210.1: 18399-21014"
6	gp42	ANT44427.1	KX171210.1: 36582-37592
7	DNA primase P4 type	ANT44428.1	KX171210.1: 37607-39775
8	gp44	ANT44429.1	KX171210.1: 39824-40558
9	virion associated RNA polymerase	ANT44439.1	KX171210.1: c44785..54981
10	16.5 kDa protein	ANT44441.1	KX171210.1: c56547-57014
11	gp54	ANT44443.1	KX171210.1: c59274-60239
12	major coat protein	ANT44445.1	KX171210.1: c60965-62164
13	gp57 (tape measure)	ANT44446.1	KX171210.1: c62199-63389
14	portal protein	ANT44448.1	KX171210.1: c63797-65977
15	30 kDa protein	ANT44452.1	KX171210.1: c67110-67745
16	terminase, large subunit	ANT44453.1	KX171210.1: c67742-69394
17	gp69	ANT44454.1	KX171210.1: c69391-70119
