# Supplementary material for: Recombination Events in Putative Tail Fibre Gene in Litunavirus Phages Infecting Pseudomonas aeruginosa and Their Phylogenetic Consequences
Source: Viruses. 2022 Nov 29;14(12):2669. doi: 10.3390/v14122669 (PMC9786124; doi:10.3390/v14122669)
Supplement: Supplementary file 1 [file viruses-14-02669-s001.zip › Table S2.pdf]

Table S2. The composition of the variable region used in the phylogenetic analyses in this study and its position in the alignment and in the genome of the selected *Litunavirus*, vB_Pae1369P-5.

Partition name		Variable region				
	Putative tail fiber protein gene					adjacent downstream gene
	N-terminal end	GrpE domain	GDSL-like lipase/acylhydrolase domain	pyocin knob domain	C-terminal end	
Position in the alignment of tail fibre DNA matrix (nt)	1-321	322-690	691-2319	2320-2847	2848-3438	3438-3990
Position in tail fibre protein gene of the phage vB_Pae1369P-5 (nt)	1-321	322-606	607-2178	2179-2688	2689-3246	-

nt - nucleotides
