# Supplementary figures and images for: Recombination Events in Putative Tail Fibre Gene in Litunavirus Phages Infecting Pseudomonas aeruginosa and Their Phylogenetic Consequences
Source: Viruses. 2022 Nov 29;14(12):2669. doi: 10.3390/v14122669 (PMC9786124; doi:10.3390/v14122669)

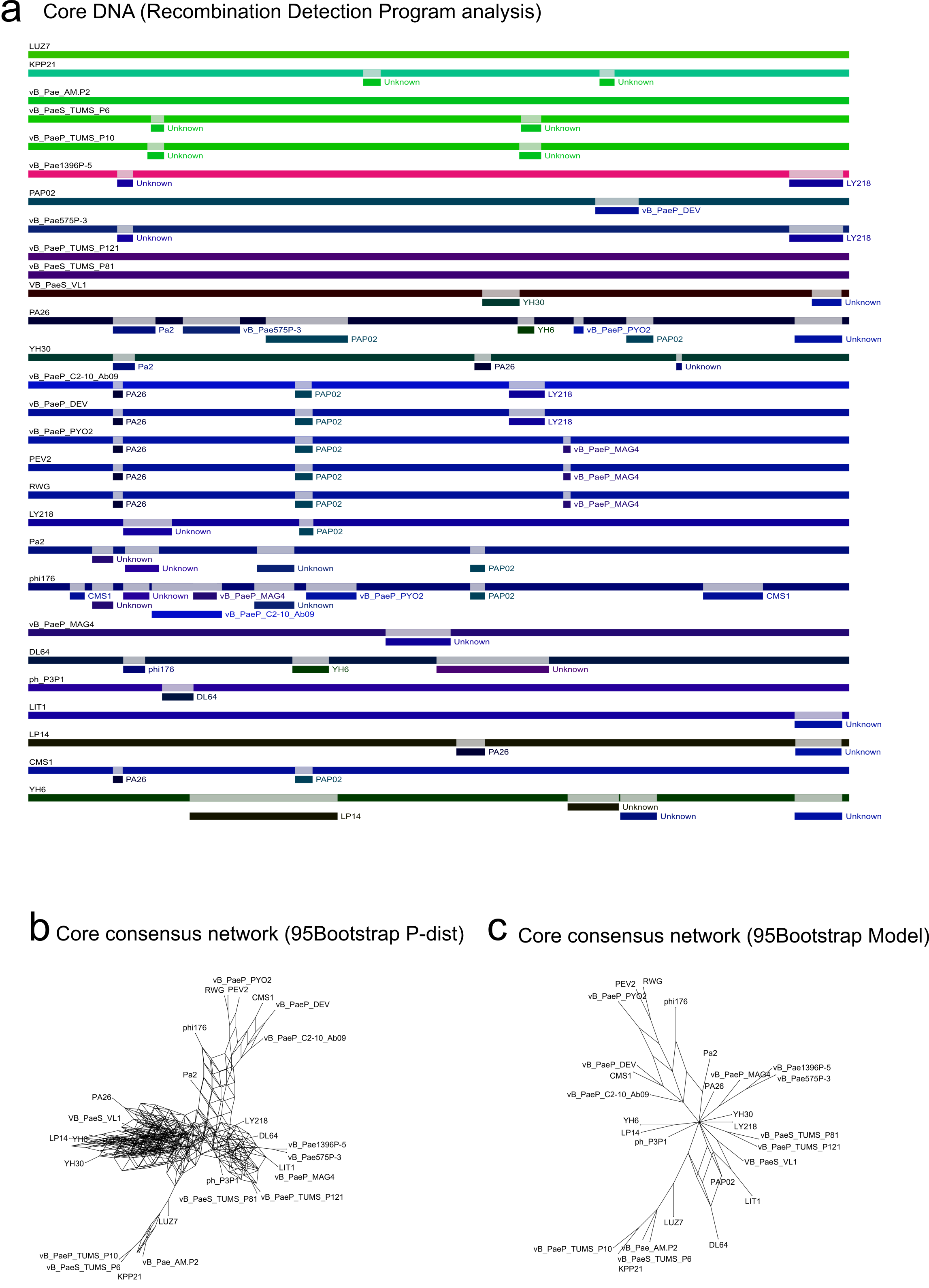

Supplement: Supplementary file 1 [file viruses-14-02669-s001.zip › Figure S1.tif]
